# Filial Care in Transition: Linguistic and Emotional Patterns in Online Discourse Among Emerging Adults in Taiwan

**DOI:** 10.3390/bs15101417

**Published:** 2025-10-18

**Authors:** Nai-Huan Hsiung, Chung-Fan Ni, Charles Silber, Justin Jacques, Cass Dykeman

**Affiliations:** 1 Department of Nursing, College of Medicine, National Sun Yat-sen University, Kaohsiung City 80424, Taiwan; 2 Department of Rehabilitation and Mental Health Counseling, College of Education, Western Oregon University, Monmouth, OR 97361, USA; 3 Department of Mathematics, College of Education, Rutgers University, New Brunswick, NJ 08901, USA; 4 Threat Assessment and Disaster Behavioral Health Service, Bolante.net, Salem, OR 97304, USA; 5 Department of Counseling, College of Education, Oregon State University, Corvallis, OR 97331, USA; dykemanc@oregonstate.edu

**Keywords:** filial piety, caregiving, social media discourse, emotional expression, corpus linguistics, intergenerational obligation

## Abstract

As Taiwan’s population ages, traditional filial piety expectations face modernization challenges, yet few studies examine how emergent adults linguistically negotiate these cultural tensions digitally. This study addresses this gap by analyzing how emerging adults in Taiwan express and reinterpret filial obligations toward aging parents through online discourse. Emerging adults represent a particularly meaningful demographic because they straddle traditional filial norms and modern independence, making their language use a valuable indicator of cultural transition. We analyzed 1976 Dcard posts from 30 discussion threads (2017–2023) using computational linguistics. LIWC-22 assessed emotional expression patterns, while Sketch Engine conducted keyness analysis and collocation mapping around filial care keywords. Posts were compared against Chinese web corpus norms. Quantitative emotion analysis revealed dominant positive emotions (M = 3.93) alongside significant negative emotions (M = 3.30), with anger and sadness exceeding broader Chinese online communication norms. Keyness analysis identified economic concerns as central themes. Collocation analysis around “filial piety” showed associations with “limits”, “willingness”, and “define”, indicating conditional rather than absolute conceptualization. Findings indicate that emerging adults in Taiwan reinterpret filial piety through reciprocal emotional bonds rather than strict hierarchical duty, negotiating traditional expectations with contemporary economic realities and personal autonomy. The implications of these findings highlight how cultural values adapt in response to modernization and digital communication, offering insight into evolving intergenerational relationships and informing future cross-cultural aging and caregiving research.

## 1. Introduction

As Taiwan’s population ages, caregiving responsibilities are increasingly falling on younger generations, raising questions about how filial obligations are understood and negotiated today (Pan et al., 2022; Pope et al., 2022). Filial piety, rooted in Confucian tradition, remains a powerful cultural norm, yet shifting family structures, personal autonomy, and socioeconomic pressures are reshaping its practice in filial care (Bedford & Yeh, 2019; Furstenberg et al., 2015; Lum et al., 2016; Wu, 2022). Studies from Mainland China have examined the evolving meanings of filial piety in response to modernization and urbanization (Lin & Wang, 2022; Yeh, 2023). In Taiwan, by contrast, the distinction between filial piety and filial care has become increasingly salient. While filial piety continues to serve as a strong cultural ideal, filial care practices are being actively renegotiated under the influence of socioeconomic pressures, shifting gender roles, and evolving policy frameworks.

Language is not only a medium that reflects cultural values but also a resource through which those values are reshaped. In digital spaces, linguistic expression can reproduce traditional filial expectations or contest them, making language a site of cultural change. Social media platforms such as Dcard thus provide a real-time lens for observing how cultural scripts of filial piety are sustained, adapted, or resisted in everyday communication (Bedford & Yeh, 2021; Caliandro, 2018). Dcard, often described as Taiwan’s equivalent of Reddit, is a topic-driven and largely anonymous platform where users (primarily college students) post and discuss a wide range of issues, from daily life to cultural debates (National Development Council, 2017). With roughly 50.8% of users aged 15–24 and over 42,000 daily visitors (C. M. Chen, 2020; National Development Council, 2017), Dcard is the country’s leading platform for young people in Taiwan. It is especially relevant for emerging adults, a developmental stage typically spanning ages 18–25 and characterized by identity exploration, self-focus, instability, and possibilities (Arnett, 2000). This demographic specificity makes it a particularly valuable site for analyzing how Taiwanese youth negotiate filial obligations at the intersection of tradition and autonomy.

Few empirical studies have analyzed the linguistic and emotional dimensions of caregiving discourse. Social media provides real-time, unfiltered data often absent from traditional survey research. This study contributes methodologically, through corpus linguistics and LIWC-based emotion analysis, and substantively, through a reinterpretation of filial piety concepts. Taiwanese emerging adults are a particularly meaningful population. They straddle the line between honoring traditional norms and pursuing independence, making their discourse a valuable indicator of cultural change (Bedford & Yeh, 2021; Charenkova, 2023; Pope et al., 2022). Although our study analyzes online discourse, the focus is not on discourse analysis in the sociolinguistic sense (e.g., constructing social reality) but on computational linguistic measures of emotional and lexical patterns. This approach captures the nuanced ways in which filial piety is reinterpreted in a multicultural society (Angle, 2022; Taiwan Council of Indigenous Peoples, 2025), where Minnan, Hakka, and Indigenous kinship logics intersect with Confucian ideals to produce hybrid caregiving norms. Such contact enables youth discourse to affirm filial duty while reframing its enactment toward reciprocity, negotiated sharing, and engagement with formal services.

### 1.1. Filial Piety: Concept and Context

Filial piety is a foundational value in East Asian societies, especially those influenced by Confucianism. It encompasses emotional and instrumental care for elders, emphasizing respect, obedience, and familial duty (Bedford & Yeh, 2019). In Taiwan, adult children are often expected to provide physical, emotional, and financial support to aging parents (Arber & Timonen, 2012; George et al., 2016). However, modernization has complicated these expectations, with smaller family sizes, urban migration, and changing economic conditions reshaping the caregiving landscape (Pan et al., 2022; Wu, 2022).

The emotional burden of caregiving is well-documented. Early research among Asian Americans found that failure to meet filial expectations often resulted in guilt, shame, and anxiety (P. N. Chen, 1982). Cross-cultural research shows that strong filial obligations correlate with increased depressive symptoms in caregivers (Pan et al., 2023), while emotional strain and identity conflict arise when traditional duties clash with personal aspirations (Y. J. Chen et al., 2022; Tsai et al., 2008; Xiao et al., 2024). Contemporary work suggests that filial piety is shifting from authoritarian models of absolute obedience to more egalitarian frameworks emphasizing pragmatic obligations and compassionate reverence (Lum et al., 2016). In many Chinese societies, filial caregiving has become contingent on adult children’s abilities and resources rather than unconditional duty (Lum et al., 2016).

### 1.2. Filial Piety, Filial Care, and Contemporary Psychological Theories

While filial piety and filial care are historically linked, they are conceptually distinct. Filial piety refers to the normative value system rooted in Confucian tradition, emphasizing respect, obligation, and moral duty (Lum et al., 2016). Filial care, by contrast, represents the enacted practices of caregiving (the emotional, financial, and instrumental support behaviors) through which individuals fulfill or negotiate filial obligations. Scholars have argued that the English term *piety* misleadingly implies religious devotion. Li (2023) instead recommended “filial care” as a more accurate translation of *xiao*, capturing its relational and pragmatic orientation. This distinction also aligns with psychological research differentiating filial attitudes from filial behaviors, highlighting the need to consider both internalized values and enacted practices (Bedford & Yeh, 2021). S. X. Chen et al. (2007) further demonstrated that filial attitudes and filial enactments are separable yet interrelated dimensions, with behaviors shaped by cultural values, social beliefs, and self-construals above and beyond attitudes. Similarly, recent frameworks conceptualize filial piety as multidimensional, encompassing beliefs, values, affect, and behaviors that evolve across sociocultural contexts and life stages (Kyeong et al., 2025). Taken together, this literature underscores the importance of distinguishing filial attitudes (value commitments), filial affect (emotional-motivational states), and filial care (behavioral enactments). Our study adopts this distinction to examine how emerging adults in Taiwan linguistically negotiate filial meanings in online discourse.

This conceptual lens resonates with identity theory, which views roles and obligations as central to the negotiation of self-concept (Burke & Stets, 2009). From this perspective, filial expectations function as identity-relevant roles that young adults may embrace, resist, or reinterpret as they transition to adulthood. Filial caregiving thus becomes not only a cultural duty but also a site of identity negotiation, where emerging adults balance traditional values with autonomy.

Filial responsibility continues to evolve as societies age and family dynamics shift (Furstenberg et al., 2015; Kalmijn, 2019). Today’s young adults face dual pressures of caring for parents while pursuing individual goals (Charenkova, 2023). Structural changes (e.g., increased mobility, shifting gender roles, rising eldercare costs) further complicate intergenerational support (Guo et al., 2019; Zhang, 2022). Although caregiving norms remain strong in Confucian and Buddhist contexts (Bedford & Yeh, 2021; Feuchtwang, 2016), younger generations increasingly favor autonomy and professional support (Charenkova, 2023; Yi & Lin, 2019). Bengtson and Roberts’s (1991) intergenerational solidarity theory highlights the persistence of family bonds through emotional connection and shared values, while Bedford and Yeh’s (2021) dual filial piety model illustrates the transition from authoritarian to reciprocal approaches. In Taiwan, modernization has accelerated this evolution, with Yi and Lin (2019) documenting a shift from strict reliance on children to broader social welfare, reflecting a more flexible understanding of filial duty that prioritizes autonomy.

This shift is especially evident in gendered caregiving expectations. Traditionally, daughters and daughters-in-law bore the bulk of caregiving (Chao & Roth, 2000). Yet rising female education, labor force participation, and family restructuring have challenged these norms (Yeh, 2023). Younger generations now negotiate caregiving in ways that both reflect persistent cultural values and incorporate shifting gender norms, making emerging adult discourse a critical lens for examining these changes.

### 1.3. Linguistic Approaches to Filial Care

Language provides a powerful tool for examining how caregiving expectations are negotiated in daily life. Digital ethnography and corpus linguistics reveal emotional and cultural meanings often missed in surveys (Bedford & Yeh, 2021; Caliandro, 2018). Platforms like Dcard capture real-time expressions of filial stress, ambivalence, and resistance, showing how traditional values are reconciled with modern realities (Y. J. Chen et al., 2022).

Language has long transmitted filial values, from classical texts to digital platforms. Concepts in the *Analects of Confucius*, such as 孝順 (*xiàoshùn*, filial piety), 照顧父母 (*zhàogù fùmǔ*, caregiving for parents), and 養兒防老 (*yǎng ér fánglǎo*, raising children for old-age support), reflect enduring ideals of duty and reciprocity (Angle, 2022). By comparing these traditional linguistic resources with emerging expressions on Dcard, this study examines how Taiwanese emerging adults reinterpret filial obligations in contemporary contexts.

We distinguish filial piety (cultural and moral values emphasizing respect and duty) from filial care (the enacted practices of emotional, financial, or instrumental support). Contemporary models highlight this shift, showing that modern filial attitudes are increasingly conditional, task-contingent, and grounded in reciprocity rather than absolute obedience (Bedford & Yeh, 2021; Lum et al., 2016). This distinction sharpens our analytical precision in examining how young adults navigate Taiwan’s changing caregiving landscape.

Through corpus linguistics analysis of keyword patterns, emotional tone, and collocational relationships in social media posts, this study investigates how filial norms are renegotiated in a generationally dynamic and multicultural society (Pope et al., 2022; Taiwan Council of Indigenous Peoples, 2025). Prior research showed filial obligations were associated with both positive and negative emotions (Guo et al., 2019; Pan et al., 2023). However, it remains unclear how emerging adults in Taiwan, many anticipating rather than directly experiencing caregiving, express these emotions online. Our study therefore investigates both positive and negative emotional responses, alongside linguistic patterns in caregiving discourse.

Given the aforenoted, four research questions (RQs) below were designed to guide the present study. These RQs were:

**RQ1:** What are the emotional levels in social media discourse among emerging adults in Taiwan discussing the obligation of taking care of parents?
**RQ2**: How do these emotional levels compare with those found in broader Chinese online communication?
**RQ3**: Which specific word usages distinguish the caregiving discourse of emerging adults in Taiwan from word usage in broader Chinese online communication?
**RQ4**: What are the collocational relationships surrounding the term “filial piety” in the online discourse of emerging adults in Taiwan compared to broader Chinese online communication?

In RQ2 and RQ3, broader Chinese online communication refers to large-scale reference corpora used as benchmarks, including a validated Chinese LIWC reference corpus and the zhTenTen web corpus (details in Section 2).

## 2. Method

### 2.1. Design

This study employed both a computer-assisted text analysis design for RQs 1–2 and a corpus linguistic design for RQs 3–4. To address RQs 1–2, we used Linguistic Inquiry and Word Count (LIWC-22) (Pennebaker et al., 2022) with the validated Chinese LIWC dictionary (Huang et al., 2012). For RQs 3–4, we used Sketch Engine, a widely adopted corpus linguistics platform, to conduct keyword and collocation analyses. Together, LIWC and Sketch Engine provide word- and category-level statistical outputs, allowing us to quantify emotional tone and lexical patterns without engaging in interpretive discourse analysis.

LIWC has demonstrated cross-linguistic validity for Traditional Chinese, including category-level equivalence in emotional constructs, as evidenced in validation studies of the CLIWC dictionary (Huang et al., 2012). The included psychological process variables regarding emotions were positive emotion and the negative emotion subcategories of anxiety, anger, and sadness (Boyd et al., 2022). These categories were selected based on their strong empirical foundation for indexing discrete emotional states in psychological research and their relevance in caregiving and health-related discourse (Huang et al., 2012; Tausczik & Pennebaker, 2010). Positive emotion captures affective tone related to gratitude and well-being, while the negative subcategories allow more granular detection of emotional stress types known to vary across caregiving experiences. LIWC’s internal consistency and validation at the category level (e.g., Huang et al., 2012; Rude et al., 2004) support their use as reliable proxies for affective content in natural language.

For the third RQ the variable was word frequencies. For the final RQ the variable was word associations. The level of measure for all variables was continuous. The unit of analysis was words. Given the public nature of the data, no human subjects review was required. The existing data is subscription-based and publicly available. All data were anonymized and publicly accessible at the time of collection; consistent with established ethical guidelines for social media research, no user-identifiable content was reported, and informed consent was deemed unnecessary due to the non-interactive, observational nature of the study (Townsend & Wallace, 2016). During the preparation of this manuscript/study, the authors used AI tools (ChatGPT 4O, 5, and Claude Sonnet 4) to brainstorm plausible reasons and organize the obtained results, proofread and edit the manuscript for grammar and APA style conformance, create figure for keyness results, and revise abstract, and title. The authors have reviewed and edited the output and take full responsibility for the content of this publication. Keyness refers to those words whose frequency more often than would be expected by chance in comparison with the reference corpus (Gabrielatos, 2018).

### 2.2. Emerging Adults Online Discussion (Study) Corpus

#### 2.2.1. Register, Scope, and Sources

This study analyzes discussions in Traditional Chinese on Dcard, Taiwan’s leading social media platform primarily used by college students and young adults https://www.dcard.tw/f (accessed on 22 May 2023). Dcard initially requires registration with a valid university or college email address in Taiwan, verifying student status at account creation. Although accounts persist after graduation, the active user base remains predominantly ages 15–24 (50.8%) with high college student participation (C. M. Chen, 2020; National Development Council, 2017). The platform allows users to post with their ID or anonymously, creating a space where emerging adults freely discuss sensitive topics including family obligations. This demographic concentration makes Dcard particularly well suited to capturing youth perspectives on filial piety.

The seed keywords were 孝順 (filial piety), 照顧父母 (caregiving to parents), and 養兒防老 (raising children for old-age support). They were deductively derived from established literature on filial piety in Taiwan (e.g., Bedford & Yeh, 2021; Yeh, 2023). Their cultural salience was further validated through consultation with two scholars specializing in family studies of Taiwan and informal feedback from graduate students. Our corpus comprises Dcard posts published between 2017 and 2023 containing these key terms related to intergenerational caregiving: To minimize confirmation bias, the seed terms themselves were not included in the interpretation of collocation results, and comparisons were made with reference corpora (zhTenTen; LIWC dictionary categories). No AI tools were used for keyword selection. This timeframe captures discussions during significant policy shifts in Taiwan that likely influenced young adults’ perceptions of family caregiving responsibilities. In 2017, Taiwan implemented the Long-Term Care Plan 2.0, which prioritized community and home-based services to support the elderly while reducing family caregiver burden (Executive Yuan, 2024). Concurrently, labor reforms introduced the “one fixed day off, one flexible rest day” policy and discussions about raising the retirement age to 65 (Executive Yuan, 2016). These labor reforms shaped how families negotiated caregiving obligations, as they directly influenced the time and resources available for adult children to care for aging parents, while also signaling broader societal debates about work–life balance and intergenerational support (C. F. Chen & Fu, 2020).

These socioeconomic and policy changes created a distinctive context for our study, as they intensified caregiving pressures on middle-aged adults while potentially reshaping young adults’ attitudes toward future caregiving responsibilities. By analyzing discourse during this period of policy transition, we can examine how emerging adults conceptualize filial obligations within Taiwan’s evolving social welfare landscape. The corpus specifically focuses on how emerging adults articulate their emotional responses to traditional filial expectations while navigating contemporary economic and social realities.

#### 2.2.2. Demographic Variables

Given the nature of how the data source is constructed, precise demographic variables could not be ascertained from the Dcard posts analyzed in this study. Additionally, generational status, cultural backgrounds, and individual users’ platform engagement patterns remained unknown due to Dcard’s anonymity protections. However, the user base consists primarily of emerging adults, reflecting the platform’s institutional origins and continued association with university communities in Taiwan (National Development Council, 2017; Digital Marketing for Asia, 2024).

#### 2.2.3. Circularity with the Scrape and Results

The seed words for the scrape to create this focused corpus (i.e., emerging adults’ discourse on caregiving of the elderly) are also likely in the results of the keyness analysis (RQ3). These seed words are the initial search terms that guided the collection of relevant social media posts for analysis. Without caution upon the part of the researcher, a circularity could enter the interpretation of the results. To counteract this threat, the seed words will not be part of the interpretation of the results for this RQ.

#### 2.2.4. Preprocessing

The authors downloaded 30 discussion threads (2017–2023) yielding 1976 posts after removing one empty post. The final corpus contained 82,156 tokens and 11,320 types (average post length = 41.6 tokens; range = 1–1590). Tokens are single occurrences of a word form in a text (Brezina, 2018). Type is a particular or unique word form (McEnery & Hardie, 2012).

Because Dcard is anonymous and posts can be authored by the same user across threads, user-level demographics cannot be reliably inferred. Therefore, we report corpus-level metrics only. A word type is a unique word form in a corpus, such as a noun (dog), verb (run), adjective (brave), or function word (the) (Brezina, 2018). We removed one empty comment with only the posting date. The final study corpus used for analysis contained 1976 posts. The average post size was 41.58 words with a range of 1 to 1590. All posts added together lead to a total corpus size of 82,156 words.

### 2.3. Reference Corpuses (Broader Chinese Online Communication)

#### 2.3.1. Reference Corpus for RQ2

The reference corpus is a LIWC dictionary Chinese version (Huang et al., 2012), which is embedded in the LIWC-2022 software. The Chinese version of the LIWC dictionary contains 30 linguistic categories and 42 psychological categories. Validity and reliability for this version is well established. Huang et al. (2012) analyzed 100 Chinese and English articles, finding high correlations between the percentages of parts of speech detected by Chinese LIWC and LIWC 2007, and that melancholic Chinese texts, consistent with English analyses using LIWC, used more first-person singular pronouns, fewer first-person plural pronouns, and more negative emotional words compared to a control group. Given the anonymous nature of the scraping, demographic variables such as gender were not available.

#### 2.3.2. Reference Corpus for RQ3

The Chinese Web Corpus (zhTenTen), built by Sketch Engine, is a large-scale corpus of approximately 2.4 billion words of web texts in Traditional Chinese collected in 2017 using standardized TenTen methods (Kilgarriff & Kosem, 2012; Kilgarriff et al., 2021). The zhTenTen corpus is not disaggregated by region and is widely used as a benchmark for online communication in Chinese. In this study we specifically drew on the Traditional Chinese version for comparison. Given the anonymous nature of the scraping, demographic variables such as gender were not available.

### 2.4. Measures

#### 2.4.1. Frequency, Type, and Token (All RQs)

The frequency count is the most basic statistical measure within corpus linguistic methodology. It is a simple tallying of the number of instances of a specific type of word that occurs in a corpus (McEnery, 2013). The definitions of Type and Token are in Section 2.2.4.

#### 2.4.2. Psychological Processes (RQs 1–2)

The psychological processes include emotion variables from the affect category; emotion encompasses: positive emotion and negative emotion (anxiety, anger, and sadness) (Boyd et al., 2022). The output is a normalized score (i.e., percentage of all words).

#### 2.4.3. Keyness (RQ3)

To identify key terms, Sketch Engine applies three primary statistical methods: Document Frequency (DOCF), Average Reduced Frequency (ARF), and Simple Maths. DOCF evaluates how widely a word is distributed across documents, helping to distinguish between corpus-wide relevance and localized repetition (Gries, 2024). ARF adjusts raw frequency to better reflect dispersion and guards against overrepresentation from long or repetitive documents. Simple Maths offers a normalized comparison of relative word frequencies between the study and reference corpora, yielding intuitive keyness scores useful for interpretability in large-scale corpora (Kilgarriff et al., 2014). These methods complement one another and are reported together to provide a nuanced view of lexical salience.

#### 2.4.4. Collocation (RQ4)

A co-occurrence relationship between words such that words are said to collocate if one is more likely to occur in the presence of the other than elsewhere (McEnery, 2013). For the present study, collocation strength was measured by Log Dice. The Log Dice coefficient is a measurement that quantifies the similarity between two sets of data, in this case, the occurrences of two words (Rychlý, 2009). LogDice = 14 + log2($\frac{2\;\times \;O_{11}}{R_{1}+C_{1}}$); where

- *O*_11_ represents the observed frequency of the collocation, i.e., the number of times the two words occur together within a defined span.
- *R*_1_ represents the total frequency of the first word in the corpus.
- *C*_1_ represents the total frequency of the second word in the corpus.

#### 2.4.5. Node Word (RQ4)

In the study of collocation, the node word is the word whose co-occurrence patterns are being studied (McEnery, 2013).

### 2.5. Apparatus

There were three apparatus employed in this study: Linguistic Inquiry and Word Count (LIWC-22) (for RQs 1–2), JASP (for RQ2) and Sketch Engine (for RQs 3–4). The LIWC software is an advanced text analysis tool used to evaluate psychological, linguistic, and emotional aspects of written texts (Tausczik & Pennebaker, 2010). The program consists of 90 analyzable output variables, focusing mainly on psychological processes. The variables are scored by comparing the percentage of words being analyzed to a dictionary of words in categories and sub-dictionaries (Smith-Keiling & Hyun, 2019). For this study, the authors used the Chinese version of the LIWC dictionary with well-established reliability and validity standards (Huang et al., 2012). All statistical analyses were performed using R graphical user interface JASP (JASP Team, 2024). Sketch Engine software is a web-based corpus linguistic analysis tool widely used in published studies (Kilgarriff et al., 2021).

### 2.6. Data Analysis

For RQs 1–2, we employed methodological procedures aligned with best practices in corpus linguistics. First, we randomized the downloaded posts to avoid clusters from the same thread, which can introduce sampling bias (Egbert & Biber, 2019). We then segmented the comments into 500-word chunks, consistent with current corpus linguistic research standards for traditional Chinese language analysis (C. H. Chen, 2023). This segmentation approach serves multiple methodological purposes: it prevents larger posts from being statistically overrepresented in the analysis of target variables, creates manageable units for consistent analysis, and contributes to the overall statistical validity of the findings (Egbert & Biber, 2019). The combined process of randomization and standardized segmentation aligns with core principles of corpus design, which emphasize the importance of balanced sampling to ensure representativeness of the linguistic phenomena under investigation (Weisser, 2016).

In terms of RQ1, descriptive statistics will be reported related to emotions in psychological processes (positive emotion, negative emotion: anxiety, anger, and sadness) using LIWC-22 (Boyd et al., 2022). Regarding RQ2, differences between study corpus and the reference corpus (Huang et al., 2012) were determined using both Frequentist and Bayesian analyses. All statistical analyses were performed using JASP. With reference to RQ3, keyword analysis comparing terms related to caregiving with Chinese web reference corpus zhTenTen with the following metric reported: raw frequency, normalized frequency, and an effect size (i.e., Simple Maths) (Kilgarriff & Rundell, 2002). This metric basically says a word is X-times (depending on the score) more frequent in corpus Y than corpus Z (Kilgarriff et al., 2021). In this article the top 20 keywords with a score 100 or greater will be considered. Concerning RQ4, raw count and a measure of association (i.e., logDice) will be provided. Using this metric to compare two scores, plus 1 point means twice as frequent collocation and plus 7 points means roughly 100 times frequent collocation (Rychlý, 2009). Only collocations with a logDice score 9 or greater will be considered. To avoid collocation results being overly influenced by “clumpiness” in certain posts no words with an ARF score below 2.0 will be considered (Venuti & Fruttaldo, 2019). Readers interested in learning more about Simple Maths and logDice can obtain detailed information on the Sketch Engine website (Kilgarriff et al., 2021). Complete records of the results for RQs 1–4 are available on this research project’s webpage: https://osf.io/qgdpw/?view_only=87beac906ef84d3d98d83ce6794508f6 accessed on 24 September 2025.

## 3. Results

### 3.1. Research Question 1 (RQ1)

The emotional expressions regarding the obligation of caring for parents on the social media of Taiwan revealed varied sentiments. In regard to RQ1, the social media users indicated a generally positive outlook ($\bar{x}$ = 3.93, SD = 1.34) towards caregiving obligations while also prominently expressed negative emotions ($\bar{x}$ = 3.30, SD = 0.99). Anxiety, anger, and sadness were less pronounced. Coefficient of variation values underscored the variability in the expression of these emotions, particularly highlighting the consistency in negative and positive emotions (CV = 0.3 and 0.34, respectively) compared to the higher variability found in anxiety, anger, and sadness (CV = 0.87, 0.77, and 0.87, respectively). Skewness measures suggested a slight positive skew for negative emotions (0.34) and more positive skewness for anxiety (1.08), anger (1.05), and sadness (0.88), indicating a tail towards higher scores for these emotions.

### 3.2. Research Question 2 (RQ2)

Before proceeding to RQ2, it is important to note that assumption checks indicated skewness; right skewed for negative emotions, anxiety, anger, and sadness suggesting more zero or low-intensity scores with fewer high-intensity scores. However, the *t*-test demonstrated fairly stringent robustness of validity, and power is not greatly affected when medium or large effect sizes were examined (Jennings et al., 2002). Therefore, we proceed with the one-sample student *t*-tests to compare observed emotion levels in our social media data against established norms from the Chinese LIWC dictionary (Huang et al., 2012). The comparison values were: positive emotions ($\bar{x}$ = 2.6, SD = 1.5), negative emotions ($\bar{x}$ = 1.3, SD = 1.2), anxiety ($\bar{x}$ = 0.3, SD = 0.4), anger ($\bar{x}$ = 0.3, SD = 0.4), sadness ($\bar{x}$ = 0.2, SD = 0.3). Significant findings across various emotions were found. Positive emotions were highly prevalent, with a large effect size (Cohen’s *d* = 1.00), indicating a strong sentiment of positivity in discussions (*t* = 12.84, *p* < 0.001). This outcome is supported by an extreme Bayes Factor (BF10 = $2.51\;\times \;10^{23}$), suggesting overwhelming evidence for the alternative hypothesis over the null. Negative emotions were markedly evident, demonstrated by a huge effect size (*d* = 2.02), significant *t*-test result (*t* = 25.93, *p* < 0.001), and an extreme Bayes Factor (BF10 = 2.29 × 10^56^), underscoring the substantial presence of negative emotions in discussions about filial piety and caregiving obligations. Conversely, anxiety showed a very small effect size (*d* = 0.05), with nonsignificant *t*-test result (*t* = 0.67, *p* = 0.507) and a moderate Bayes Factor (BF01 = 9.27), indicating moderate evidence for the null hypothesis, suggesting that anxiety is not a predominant emotion in this discourse. Anger and sadness were present with medium (*d* = 0.57) and small (*d* = 0.44) effect sizes, respectively, both showing significant *t*-test result (anger: *t* = 7.28, *p* < 0.001; sadness: *t* = 5.65, *p* < 0.001) and extreme Bayes Factors (anger: BF10 = 5.85 × 10^8^; sadness BF10 = 147,037.8), indicating strong evidence for these emotions.

### 3.3. Research Question 3 (RQ3)

First, seed words and words that direct synonyms/antonyms or overlapping concepts were identified to address circularity concerns. These words were:

1. 孝順 (Filial piety)—Directly matches the seed word.
2. 養兒 (Raising children)—Overlaps directly with 養兒防老, representing a part of the phrase.
3. 孝養 (Filial support)—A close synonym of 孝順 with a similar meaning of filial piety.
4. 孝親費 (Filial duty expenses)—Related concept to 孝順, directly involving the practical side of fulfilling filial obligations.
5. 防老 (Support of aging/Protecting oneself in old age)—Closely tied to 養兒防老 in terms of ensuring well-being during old age.
6. 扶養 (Providing support)—Overlaps with 照顧父母, as it involves caring for or supporting one’s parents.
7. 不孝 (Unfilial)—Conceptually tied to 孝順 as it represents the opposite of fulfilling filial duties.

The remaining 13 words will be the focus of the analysis. The three highest-scoring non-seed words were 們 (*They/Them*, simple maths = 360.22), 療養院 (*Nursing home/Convalescent home*, simple maths = 181.89), and 媽媽 (*Mom/Mother*, simple maths = 151.81). See Table 1 for complete results from the 20 words with the strongest keyness scores.

### 3.4. Research Question 4 (RQ4)

**Figure 1:**
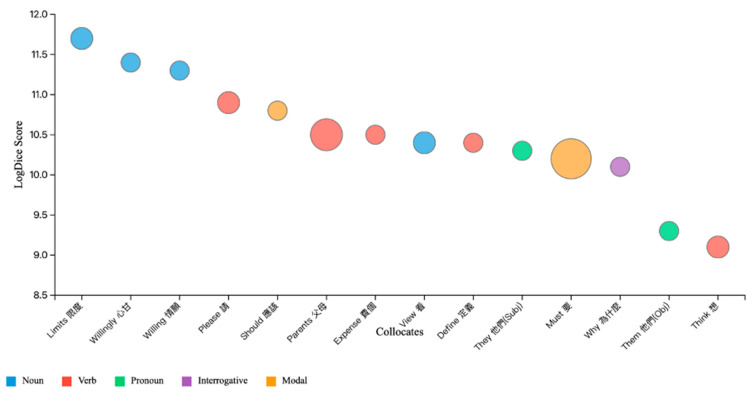
Collocates of the Node Word 孝順. Note. Bubble size represents frequency of collocation. Collocates are ordered by logDice score (Y-axis). Colors indicate part of speech classification. Data derived from Sketch Engine corpus analysis with minimum logDice threshold of 9.0.

Sketch Engine assigned general parts of speech (PoS) to all collocates. While PoS parsing is a well-established research practice, it comes with limits. As Firth (1968) famously noted, “You shall know a word by the company it keeps!” (p. 13). Thus, to understand fully what 孝順 (filial piety) means in the natural language of emerging adults, we identified words that were close neighbors (i.e., collocates) to this word. The three strongest collocates were: (1) 限度 (Limit), logDice = 11.7, (b) 心甘 (Willingly), logDice = 11.4, and (c) 情願 (Willing), logDice = 11.3. Figure 1 presents the 14 strongest collocates.

The initial Sketch Engine analysis classified collocates using automated PoS tagging, which provided a basic distinction between nouns and verbs. Upon closer examination of the Traditional Chinese linguistic texts, the need for a more granular classification was needed given how emerging adults construct meaning around filial piety. For example, 心甘 “willingly,” initially tagged as a noun. However, reviewing the usage of this word pointed to it functioning more as an adverbial phrase. This revision was critical because many high-scoring collocates captured conditional or voluntary orientations [e.g., 限度 (limits), 心甘 (willingly), 情願 (willing), 應該 (should)] rather than authoritarian imperatives.

The syntactical transition of terms in this corpus like 心甘 (“willingly”) and 情願 (“willing”) appear in noun categories even though their contextual meanings clearly function as expressions of voluntary care. These collocates resonate with representative posts in which users described caregiving not as forced compliance but as an emotionally meaningful act. For instance: 「...負起教養的責任，子女也會愛父母，心甘情願供養父母，這是愛的流動」*(…bear the responsibility of nurturing and educating, children will also love their parents, willingly and gladly supporting them; this is the flow of love).* Such examples show that willingness and affection are explicitly foregrounded in discourse, reinforcing the interpretation that filial piety is framed by emerging adults as negotiated, context-dependent, and voluntary rather than unconditional obedience.

## 4. Discussion

This study explored how Taiwanese college students and young adults discuss their responsibilities toward parental caregiving on the social media platform Dcard. Specifically, we examined emotional expressions toward filial piety and caregiving, comparing these to normative reference groups, and analyzed key linguistic patterns that reveal emerging attitudes toward traditional caregiving obligations. Our findings demonstrated both continuity and evolution in how emerging adults in Taiwan conceptualize filial responsibilities within contemporary social and economic contexts. These results underscore that while filial piety is largely viewed through a culturally positive lens, it is also accompanied by significant emotional ambivalence. Below, we interpret our findings and offer possible explanations for the results, organized by each research question (RQ).

### 4.1. Emotional Expression in Caregiving Discourse (RQ1)

LIWC analysis revealed that Dcard users expressed both positive and negative emotions when discussing caregiving. High positive emotion scores (M = 3.93) likely reflect the cultural valorization of caregiving as a moral obligation rooted in Confucian norms (Su-Kubricht & Miller, 2023; Wong & Chau, 2006), though this positivity may also reflect social desirability or conformity to cultural expectations (Grimm, 2010). Comparable findings have been reported in Hong Kong, where younger caregivers often describe pride and meaning in their roles, supported by family or institutions (Shum & Lum, 2020).

Negative emotions (M = 3.30) were also present, with moderate levels of anger and sadness. These likely reflect the structural pressures associated with caregiving, including financial strain, career disruption, and perceptions of intergenerational inequality (Guo et al., 2019; Kayaalp et al., 2020; Maestas et al., 2024; Nguyen et al., 2021; Wang & Huang, 2023). Meanwhile, the overall negativity is moderate compared to typical social media discourse on emotionally charged topics (Huang et al., 2012). This suggests a nuanced emotional landscape, rather than a framing that is wholly positive or negative.

The consistently low frequency of anxiety-related words is particularly noteworthy. Rather than framing caregiving in terms of personal worry or psychological distress, emerging adults emphasized obligation, ambivalence, and conditional willingness. Several explanations may account for this pattern: cultural norms that discourage overt displays of anxiety; the use of alternative affective registers such as guilt, frustration, or resistance (Su-Kubricht & Miller, 2023; Wong & Chau, 2006); the anticipatory nature of the sample, in which many posts reflected hypothetical rather than lived caregiving experiences; and Taiwan’s cultural normalization of caregiving roles, where stress is perceived as manageable or expected (Grimm, 2010). Together, these findings suggest that emotions surrounding filial care are expressed less as personal vulnerability and more as culturally situated negotiations of obligation and reciprocity (Guo et al., 2019; Pan et al., 2023).

### 4.2. Comparison to Broader Chinese Online Communication Norms (RQ2)

In comparing emotional tone in social media discourse with established broader Chinese Online communication norms, our findings revealed that Dcard users expressed significantly higher levels of both positive and negative emotions than those observed in general online communication. Specifically, anger and sadness were elevated among Dcard users compared to the zhTenTen corpus, while anxiety levels showed no notable differences. Overall, caregiving discourse on Dcard was more emotionally intense and positively valenced than what is typically found in broader internet communication (C. M. Chen, 2020).

These differences reflect contrasts between caregiving discussions and general online discourse, rather than direct evidence of cultural change. While higher positivity and lower anxiety suggest that filial caregiving is framed differently from other online topics, this finding alone does not demonstrate evolving filial discourse. Instead, it establishes a baseline for the distinctive emotional register associated with filial piety among emerging adults. When considered alongside linguistic analyses (RQ3 and RQ4), these results provide insight into how filial norms are expressed and negotiated.

Two factors may help explain this heightened emotionality. First, many users discussed caregiving in anticipatory rather than experiential terms, projecting ideals, anxieties, or frustrations without the moderating influence of lived caregiving experience (Papacharissi, 2015; Pickett et al., 2024; Suler, 2004). Second, platform affordances likely amplified expression. Dcard’s anonymity and moderation policies create a psychologically safe space that fosters both authentic and intense emotional disclosures (Y. C. Chen, 2023; Van der Zee & Bülow, 2022). Together, these dynamics may account for the substantial effect sizes observed.

These findings support our expectation that emotional responses of emerging adults in Taiwan discussing filial piety would differ from those of the general population (Diefenbeck et al., 2017; Garas et al., 2012). While younger users remain emotionally engaged with caregiving obligations, they express both reverence and ambivalence at heightened levels, reflecting an evolving discourse where filial piety retains salience but is increasingly interpreted through reciprocity and relational autonomy (Bedford & Yeh, 2021). This reinterpretation may also be informed by early-life exposure to caregiving models: many youth in Taiwan grow up in three-generation households, observing their parents’ care for elders, which shapes their attitudes toward intergenerational responsibility (Liu et al., 2011).

### 4.3. Lexical Patterns Revealing Socioeconomic Pressures (RQ3)

Our keyword analysis comparing Dcard discourse to broader Chinese online communication (zhTenTen) revealed significant lexical patterns that illuminate how economic considerations shape attitudes toward elder care. Specifically, keywords such as 階級複製 (class/social reproduction) and 安養院 (nursing home) were significantly overrepresented, suggesting that economic pressure is not peripheral but central to how emerging adults in Taiwan conceptualize caregiving obligations. These lexical patterns provide strong, data-driven evidence of the socioeconomic stress embedded in filial caregiving discourse. The keyword “social reproduction” often appeared in comments where authors linked filial obligations to the continuation of structural inequalities. Posts reflected concerns that caregiving duties fall unequally along gender and class lines, thereby reproducing traditional social hierarchies. In this context, filial piety was discussed not just as a cultural value but also as a practice through which broader social reproduction occurs.

Additional keywords, including 威 (e.g., 威脅 *threat*, 威嚇 *intimidation*) and 扛負起 (shoulder responsibility), indicate that caregiving is frequently framed in terms of burden, constraint, or imposed duty. These linguistic markers align with prior research on youth economic precarity and intergenerational expectations in Taiwan (Levine, 2024). They also reinforce the notion that caregiving discourse is shaped by a struggle to reconcile personal autonomy with familial obligation, especially under structural conditions such as high youth unemployment and widening class disparities.

While these lexical choices offer compelling insight into the internal conflict emerging adults in Taiwan may experience, we acknowledge that such interpretations remain inferential. Without self-reported data, the link between word use and individual psychological experience cannot be confirmed. However, the consistency and salience of these economic and burden-related terms provide strong indirect support for the hypothesis that emerging adults perceive caregiving as a site of tension, where traditional expectations increasingly clash with modern financial and social realities.

### 4.4. Reinterpreting Filial Piety (RQ4)

Our collocation analysis around “filial piety” (孝順) revealed a fundamental reinterpretation of this concept by emerging adults in Taiwan, who are actively reconstructing filial piety contingent, voluntary, and reciprocal rather than absolute and mandatory. The collocate 限度 (limits, logDice = 11.7) signals a decisive break from traditional conceptualizations, with emerging adults explicitly reframing filial piety as contingent upon circumstances, relationships, and personal capacity rather than unconditional. The presence of collocates 情願 (willing, logDice = 11.3) and 請 (request, logDice = 10.9) demonstrate how emerging adults in this study reinterpreted filial care as fundamentally voluntary, something one chooses to do rather than must do. The modal distinctions further illustrate this reinterpretation: while 要 (must, logDice = 10.2, frequency = 14) still appears, it coexists with the more negotiable, 應該 (should, logDice = 10.8, frequency = 2) indicates weaker, more negotiable obligation. All this may indicate that emerging adults employ graduated modal language when discussing filial care, suggesting that emerging adults are reconstructing filial piety as existing along a spectrum of voluntary commitment rather than absolute obligation.

In sum, the collocation analysis reveals that emerging adults conceptualize filial piety as possessing a contingent and reciprocal nature that differs markedly from previous generations’ understanding. Bedford and Yeh’s (2021) dual filial piety model provides essential theoretical grounding for understanding this active reinterpretation process. Their model distinguishes between authoritarian (duty-based) and reciprocal (emotion-based) components of filial piety, with our data clearly showing a shift toward the latter. These results echo patterns identified in studies from Mainland China where filial piety is increasingly framed through emotional reciprocity and mutual care (Lin & Wang, 2022). Meanwhile, the discourse in Taiwan demonstrates a more deliberate emphasis on negotiation and autonomy. The linguistic evidence reveals not merely a shift but a conscious reframing of filial piety as reciprocal, contingent upon mutual respect, emotional connection, and balanced exchange rather than one-way obligation. Terms like 情願 (willingness) and 限度 (limits) signal this reciprocal reinterpretation, where filial care becomes conditional upon the quality of family relationships and the reciprocal nature of care exchanges. This reinterpretation represents a profound cultural transformation where emergent adults in Taiwan are actively reconstructing filial piety to align with contemporary values while maintaining cultural legitimacy. As Yeh (2023) observed, authoritarian elements of filial piety are declining while reciprocal aspects transcend, but our data suggests this is not passive cultural drift, it is intentional reinterpretation.

This theoretical framework helps explain why Dcard users express both positive and negative emotions toward caregiving. Emerging adults are strategically redefining filial piety as voluntary care contingent upon reciprocal family dynamics, allowing them to maintain cultural identity while asserting personal agency. This reinterpretation transforms caregiving from burdensome obligation into voluntary, reciprocal engagement that honors both cultural heritage and individual autonomy. The result is a reconceptualized filial piety that is conditional, chosen, and mutually beneficial rather than absolute, imposed, and one-sided.

### 4.5. Theoretical Integration: Identity Development and Role Negotiation

Our findings align with broader psychological theories of identity and development, extending beyond the cultural framework of filial piety. The linguistic patterns observed in Dcard discourse reflect Arnett’s (2000) theory of emerging adulthood, in which individuals aged 18–25 explore identities while balancing competing role expectations. The collocation of 孝順 (filial piety) with terms such as 限度 (limits) and 定義 (define) illustrates this negotiation, as young adults reinterpret inherited cultural scripts in light of self-determined values.

This process also resonates with identity theory. Stryker (1980) emphasized that individuals hold multiple identities organized in a hierarchy of salience; for emerging adults in Taiwan, the filial child identity competes with emerging autonomous adult identities, producing the ambivalence captured in our LIWC analysis. Economic references, such as 階級複製 (class reproduction), further illustrate what Burke and Reitzes (1991) described as structural identity conflict, in which role expectations exceed available resources and create psychological strain.

The shift in discourse toward reciprocal rather than authoritarian filial piety reflects identity modification; adapting role meanings to maintain coherence between cultural commitments and personal autonomy. Taken together, these patterns suggest that the evolution of filial caregiving in Taiwan reflects not only cultural change but also broader developmental processes of identity consolidation under conditions of rapid modernization. This theoretical integration highlights how emerging adults in collectivistic contexts reinterpret traditional roles in ways that preserve cultural continuity while negotiating new forms of autonomy.

### 4.6. Gender Norms and Caregiving Discourse

Although not explicitly coded in our analysis, deep-seated gender norms likely shape both the emotional tone and discursive framing of caregiving among emergent adults in Taiwan. Traditional Chinese family structures have long assigned caregiving duties disproportionately to women (Zhan & Montgomery, 2003), and these cultural expectations continue to influence how individuals articulate emotional responses in caregiving contexts (Pharr et al., 2014). Research suggests that men are often socialized to maintain emotional stoicism and emphasize competence and provision, while women are more likely to express emotions tied to relational concern and moral duty (Chaplin, 2015; Timmers et al., 1998).

Such emotional norms may result in men discussing caregiving in more abstract or instrumental terms, whereas women may voice ambivalence, emotional labor, or distress more openly (Calasanti & Bowen, 2006; Kramer & Lambert-Shute, 2010). This divergence reflects broader cultural scripts that assign expressive roles to women and affective control to men, particularly within the Confucian filial framework (Stoppard & Gunn Gruchy, 1993). The gendered emotional landscape is further shaped by masculine honor ideals; men who endorse these beliefs tend to attribute more negative emotions to caregiving due to perceived threats to peer status (Gul & Uskul, 2019).

Women, by contrast, are often expected to show warmth and responsiveness to others’ needs, while men are encouraged to display positive emotion only when it reinforces independence or achievement (Stoppard & Gunn Gruchy, 1993). This can lead to contrasting emotional disclosures in digital settings, such as Dcard, where women may narrate caregiving stressors in detail, while men may understate emotional burden or frame it in utilitarian terms (Sharma et al., 2016). Additionally, ambivalent sexism, both hostile and benevolent, can influence evaluations of how men and women conform to or deviate from traditional caregiving roles (Gaunt, 2013).

Together, these dynamics suggest that gendered emotional expectations shape not only how caregiving is experienced, but how it is publicly discussed and linguistically encoded in peer-based online discourse. Future research could benefit from a gender-segmented emotional analysis to further explore these expressive asymmetries.

### 4.7. Limitations

While this study offers valuable insight into how emerging adults in Taiwan navigate filial caregiving norms, several limitations should be noted. First, the sample is drawn exclusively from the Dcard platform, which primarily attracts university students. This likely skews the data toward educated, urban, and middle- to upper-middle-class youth, underrepresenting rural, lower socioeconomic, and older populations. Additionally, Dcard’s anonymous format limited access to demographic variables such as gender, family structure, caregiving experience, and regional background. As a result, the findings may not fully reflect the broader spectrum of Taiwanese society.

Second, the corpus size was restricted to posts collected between 2017 and 2023 using only three seed terms. While this approach ensured relevance, the selected keywords, particularly “filial piety” (孝順), carry broad and context-dependent meanings, which may introduce ambiguity in scraping and interpretation. A larger and more diverse dataset, coupled with refined keyword strategies such as semantic field mapping, could improve the representativeness and analytical depth of future studies.

Third, participation on Dcard requires digital literacy, internet access, and engagement with caregiving discourse. This introduces sampling bias, potentially excluding marginalized groups less active online, such as older adults or rural residents who may hold different views on filial obligations.

Fourth, while LIWC provides a robust tool for quantifying emotional tone, it is limited in detecting culturally specific nuances, such as irony, sarcasm, or rhetorical strategies prevalent in online communication. As such, some emotional expressions, particularly those embedded in Taiwanese digital vernacular, may be misinterpreted or overlooked. Future research should complement automated analyses with qualitative methods to better capture these subtleties.

Fifth, youth discourse often involves hybrid linguistic forms, including urban slang and adolescent heteroglossia (Rampton, 2011), which standardized tools like LIWC or Sketch Engine may not fully accommodate. To better reflect the evolving linguistic landscape, researchers should consider expanding their analytic frameworks and data sources to capture a wider array of voices and expressions.

Finally, while zhTenTen provides a large-scale benchmark for general online discourse in Traditional Chinese, it cannot be cleanly disaggregated by region (e.g., Taiwan versus other Sinophone areas), which poses a limitation in attributing findings to Taiwanese online discourse alone.

### 4.8. Implications

The shift toward reciprocal filial piety revealed in this study has important implications for clinical practice, research, and policy development.

#### 4.8.1. Implications for Clinical Practice

Practitioners should tailor therapeutic approaches to acknowledge evolving filial norms rather than assuming strict hierarchical family structures. Clinicians should utilize strengths-based approaches that honor filial piety’s cultural importance while creating space for negotiating contemporary expressions (Hsu & Wang, 2011), facilitate intergenerational dialogue about caregiving expectations (Tsai et al., 2008), and design interventions addressing the emotional ambivalence emergent adults experience when reconciling cultural values with practical concerns (Khalaila & Litwin, 2011). Additionally, clinicians should acknowledge how rising demand for long-term care services challenges traditional caregiving ideals (Liu & Huang, 2009).

Our collocation analysis revealed emotionally meaningful terms such as “support,” “accompany,” and “understand” clustering around caregiving references. Therapeutic framing that highlights relational fulfillment over obligation may reduce guilt and increase perceived agency among emergent adults (Pharr et al., 2014). Culturally adapted psychoeducational materials should validate reciprocal models of caregiving as legitimate expressions of filial piety (Yeh et al., 2013).

#### 4.8.2. Implications in Research

Future research should analyze subgroup variations across four dimensions: (1) emotional expression patterns to uncover distinct coping mechanisms; (2) gender differences, particularly why more females discussed filial piety online and whether traditional caregiving expectations continue falling disproportionately on women; (3) ethnic and cultural variations among Taiwan’s ethnic groups (Tai, Hakka, and Minnan); and (4) platform comparisons examining how different social media characteristics shape emotional expression regarding filial obligations. Machine learning techniques could classify linguistic patterns reflecting emotional strain and class-based pressures to develop emotion-informed typologies of caregiving discourse.

#### 4.8.3. Implications for Policy

Taiwan’s Long-Term Care 2.0 Plan reflects ongoing efforts to address caregiving challenges created by urbanization and increasing life expectancy (C. F. Chen & Fu, 2020). However, younger generations are redefining caregiving through emotional reciprocity rather than strict familial obligation (Bedford & Yeh, 2021), while facing economic pressures that heighten mental health concerns (Su-Kubricht & Miller, 2023) and lead to prolonged co-residence patterns (Y. J. Chen et al., 2022).

Policymakers should frame formal eldercare services as emotionally compatible with filial values rather than replacements for family care, develop public messaging acknowledging the evolution toward reciprocal filial piety, create urban elder care centers emphasizing family involvement, and implement financial support programs addressing economic constraints and concerns about class reproduction identified in our keyword analysis. By aligning eldercare policy with emotional and relational themes found in our collocates such as “respect,” “compassion,” and “ease,” official discourse can reframe formal care as an extension of filial values, reducing cultural resistance and promoting inclusive caregiving structures.

### 4.9. Conclusions

This study combines computational linguistic analysis with cultural theory to examine evolving attitudes toward filial piety. By applying corpus linguistics, LIWC analysis, and collocation networks to social media discourse, we demonstrate how digital humanities approaches can reveal nuanced sociocultural shifts hidden in traditional survey research.

Taiwan’s emergent adults are actively reinterpreting filial piety through frames of reciprocity, emotional connection, and voluntary commitment. This represents not cultural erosion but cultural adaptation. The findings highlight how emerging adults navigate filial expectations within a hybrid cultural framework where traditional norms coexist with modern values of autonomy and reciprocity, offering promising pathways for understanding how culture, language, and emotion intersect in the digital age.

## Figures and Tables

**Table 1:** Keyness Results (RQ3).

	Frequency		Relative Frequency		Relative DOCF		ARF	
Item	Study	Reference	Study	Reference	Study	Reference	Study	Score
孝順 Filial piety	65	9774	2313.58	3.28	16.26	0.10	25.09	540.44
防老 Support of aging	19	1919	676.28	0.64	7.32	0.02	9.18	411.84
你們 You	26	4680	925.43	1.57	6.91	0.06	13.78	360.22
養兒 Rasing children	18	2808	640.68	0.94	6.91	0.03	8.66	330.23
扶養 Providing Support	33	8440	1174.59	2.83	8.13	0.07	11.09	306.56
孝親費 Filial duty expenses	8	144	284.75	0.05	2.03	0.00	3.18	272.57
療養院 Nursing home	8	1700	284.75	0.57	2.03	0.02	3.46	181.89
媽說 As mother said	9	3325	320.34	1.12	2.85	0.04	6.37	151.81
阿公 Grandfather	39	24,293	1388.15	8.16	5.28	0.17	7.00	151.67
了不起 Awesome	4	0	142.37	0.00	1.63	0.00	2.54	143.37
不孝 Unfilial	9	3820	320.34	1.28	3.66	0.04	5.21	140.75
階級複製 Social reproduction	4	64	142.37	0.02	1.22	0.00	2.13	140.36
爸 Father	80	57,872	2847.48	19.44	10.57	0.45	27.94	139.38
安養院 Elder care center	7	2615	249.15	0.88	2.03	0.02	2.15	133.18
戚 Kin	4	295	142.37	0.10	1.22	0.00	2.41	130.45
給家裡 Give something to family	4	388	142.37	0.13	1.22	0.01	2.93	126.84
他們 They	94	81,667	3345.79	27.43	21.14	0.89	48.43	117.72
阿嬤 Grandmom	24	19,495	854.24	6.55	4.47	0.16	9.11	113.31
合十 Palms together	8	4825	284.75	1.62	2.44	0.05	3.61	109.04
奶奶的 Damn	3	0	106.78	0.00	0.81	0.00	2.00	107.78

## Data Availability

The data is available on the research project’s webpage: https://osf.io/qgdpw/ accessed on 24 September 2025.
